# Expressions of Immune Prophenoloxidase (proPO) System-Related Genes Under Oxidative Stress in the Gonads and Stomach of the Mud Crab (*Macrophthalmus japonicus*) Exposed to Endocrine-Disrupting Chemicals

**DOI:** 10.3390/antiox13121433

**Published:** 2024-11-21

**Authors:** Ji-Hoon Kim, Kiyun Park, Won-Seok Kim, Ihn-Sil Kwak

**Affiliations:** 1 Department of Ocean Integrated Science, Chonnam National University, Yeosu 59626, Republic of Korea; jihoon4824@naver.com; 2 Fisheries Science Institue, Chonnam National University, Yeosu 59626, Republic of Korea; ecoblue8@gmail.com (K.P.); csktjr123@gmail.com (W.-S.K.)

**Keywords:** crustacean, immune defense system, mRNA expression, organ specificity, endocrine disrupting chemicals (EDCs)

## Abstract

Endocrine-disrupting chemicals (EDCs) significantly damage biological systems related to reproductive, neurological, and metabolic functions. Approximately 1000 chemicals are known to possess endocrine-acting properties, including bisphenol A (BPA) and di(2-ethylhexyl) phthalate (DEHP). This study primarily focuses on the potential effects of EDCs on the transcriptional levels of innate immune prophenoloxidase (proPO) system-related genes under oxidative stress in the gonads and stomach of the mud crab *Macrophthalmus japonicus*, an indicator species for assessing coastal benthic environments, when exposed to 1 µg L^−1^, 10 µg L^−1^, and 30 µg L^−1^ BPA or DEHP. After EDC exposure, the expression of lipopolysaccharide and β-1,3-glucan-binding protein (LGBP), a pattern recognition protein that activates the proPO system, was upregulated in the stomach of *M. japonicus*, whereas LGBP gene expression was downregulated in the gonads. In the gonads, which is a reproductive organ, EDC exposure mainly induced the transcriptional upregulation of trypsin-like serine protease (Tryp) at relatively low concentrations. In the stomach, which is a digestive organ, LGBP expression was upregulated at relatively low concentrations of EDCs over 7 days, whereas all proPO system-related genes (LGBP, Tryp, serine protease inhibitor (Serpin), and peroxinectin (PE)) responded to all concentrations of EDCs. These results suggest that the antioxidant and immune defense responses of the proPO system to EDC toxicity may vary, causing different degrees of damage depending on the tissue type in the mud crab.

## 1. Introduction

The leaching of pollutants into terrestrial and aquatic environments constitutes a global concern due to the potential risks they pose to living organisms [1,2]. Endocrine-disrupting chemicals (EDCs) are exogenous substances that either stimulate or inhibit endogenous hormonal responses, interfering with the reproduction and development of organisms [3]. Most EDCs, such as atrazine, bisphenol A (BPA), triclosan, and phthalate esters, are synthetic compounds resulting from human activities [4]. However, phytoestrogens (plant-derived) and mycoestrogens (fungi-derived) are naturally occurring substances. These synthetic compounds adversely affect organisms by inhibiting normal hormones and by mimicking endogenous hormones, leading to alterations in hormone synthesis and patterns [5,6]. BPA and di(2-ethylhexyl) phthalate (DEHP), both derived from plastics, are commonly used in household products, cosmetics, and medical devices [7,8,9,10]. BPA, at concentration of 1 mg L^−1^, has been found to induce approximately an 80% reduction in the growth rate of the marine green algae *Tetraselmis suecica* [11], whereas the survival rate of *Daphnia magna* has been shown to decrease to approximately 30% when exposed to 10 mg L^−1^ of BPA for 2 days [12]. In trout, exposure to DEHP has been found to trigger a decrease in glutathione content and to significantly disrupt antioxidant and immune defense functions [13]. Notable variations in baseline concentrations of BPA and DEHP have been observed across different regions. In the East China Sea, the concentration of BPA in sediments has been found to range from 2.2 ng g^−1^ dw to 34 ng g^−1^ dw, whereas, in water samples, BPA levels reached significantly higher concentrations [14]. The concentrations of BPA in Japan, South Korea, and India have been shown to reach quantities of 325 ng L^−1^, 141 ng L^−1^, and 1390 ng L^−1^, respectively [15]. For DEHP, concentrations in the coastal waters of China have been found to range from 60 ng L^−1^ to 617 ng L^−1^, while, in the Mediterranean Sea, levels have been shown to vary between 103 ng L^−1^ and 297 ng L^−1^ [16,17]. A notably high DEHP concentration of 45.73 µg g^−1^ has been reported in sediment from the Yellow River [18]. Although environmental EDC pollution has been linked to various adverse effects on living organisms, the impacts of EDCs on marine environments remain largely unexplored.

EDCs not only disrupt the endocrine system, but also negatively affect the immune system’s ability to defend the organism against external toxins [19]. Immunity is a biological system that protects an organism from harmful substances, including viruses, parasites, and pathogens [20]. In general, the immune system consists of two main components: the innate immune system and the adaptive immune system [21]. Innate immunity is the first line of defense against invading pathogens and includes the prophenoloxidase (proPO) system, antimicrobial peptides (AMPs), phagocytosis, and hemocyte nodulation [22]. The proPO system is activated by pattern recognition proteins (PRPs) that bind to β-1,3-glucans (*βGBP*), lipopolysaccharides (*LGBP*), and peptidoglycans (*PGBP*). These PRPs activate the proteinase, which triggers the serine proteinase cascade, leading to the activation of prophenoloxidase-activating proteinase (PAP). In turn, PAP converts proPO into phenoloxidase, which then synthesizes melanin [23]. Melanin deposition is a signal response to physical tissue damage or to the invasion of foreign substances in invertebrates [24]. The proPO system includes various genes, such as serine protease inhibitor (*Serpin*), trypsin-like serine protease (*Tryp*), and peroxinectin (*PE*) [8]. Numerous environmental factors have been linked to changes in the immune response and expression of genes associated with the proPO system. For example, the innate immunity of the blue mussel (*Mytilus edulis*) has been found to experience inhibition following changes in salinity and exposure to ZnO nanoparticles [25]. Additionally, the antibiotic sulfamethoxazole has been found to cause decreased expression levels of innate immunity-related genes, such as Janus kinase, astakine, and proPO, alongside reduced activities of antioxidant enzymes [26].

**Figure 1:**
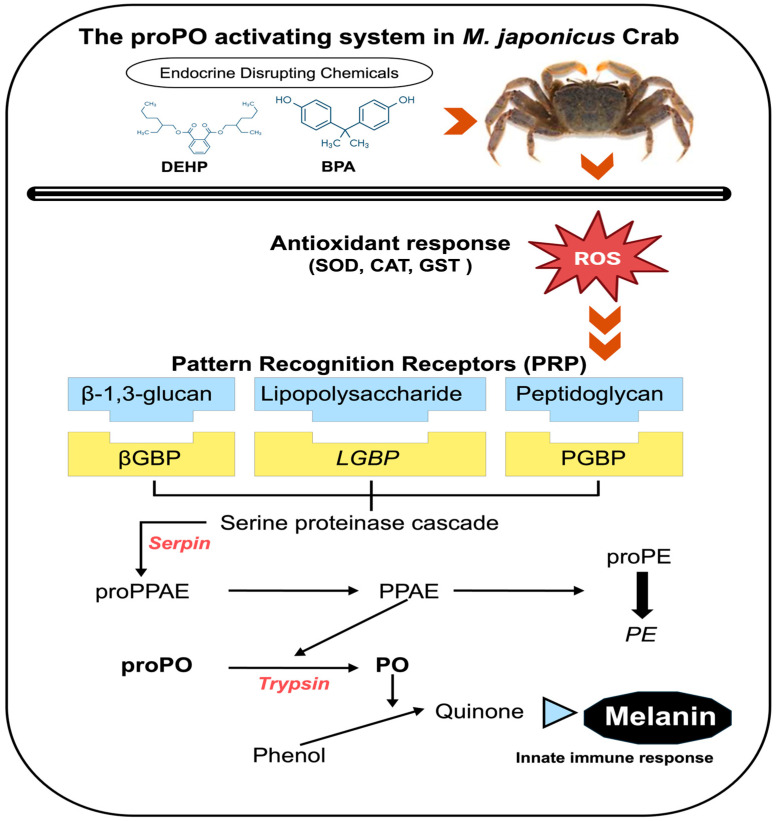
Schematic overview of the innate immune prophenoloxidase (proPO) activation system in *M. japonicus* after exposure to EDCs, including BPA and DEHP. PE: peroxinectin; proPE: properoxinectin; PPAE: proPO-activating enzyme; proPPAE: proproPO-activating enzyme. Italicized gene names indicate the genes tested in the proPO system in this study. The schematic figure was modified based on the literature [8,34].

*Macrophthalmus japonicus* is a marine benthic crab that inhabits tidal flats and is widely distributed across East Asia, including Korea, China, and Japan [27]. These crabs typically live in burrows and feed on microorganisms or organic matter on the mud surface [28]. Crabs living in tidal flats play a crucial role in the marine ecosystem’s food chain. Depending on their developmental stage, *M. japonicus* larvae feed on rotifers, diatoms, and organic matter, representing a significant food source for organisms higher up in the food chain [29,30]. They also contribute to the aeration of the mudflats through their burrowing behavior and help purify the water by consuming organic matter [31]. Gene expression analysis is commonly used to assess the toxic effects of chemical pollutants, including EDCs. Changes in gene expression can reflect biological responses to toxicity and are identified by assessing mRNA levels of antioxidant, immune, and stress-related genes in organisms. Increased mRNA expression levels of antioxidant enzymes have been observed in *Litopenaeus vannamei* exposed to polystyrene nanoplastics [32]. Exposure to the marine pollutant phenanthrene (PHE) has been shown to reduce blood cell counts and suppress immune function in the crustacean *Scylla paramamosain* [33]. In this study, we investigate the different toxic effects of EDC exposures on the immune defense system, focusing on digestive and reproductive organs in marine organisms. Particularly, we assess changes in the expression of innate immune proPO-related genes in the stomach and gonads of *M. japonicus* exposed to BPA and DEHP at varying concentrations (Figure 1).

## 2. Materials and Methods

### 2.1. Test Organism

*M. japonicus* individuals (width: 40 ± 5 mm; height: 35 ± 5 mm; weight: 8.0 ± 2.0 g) were collected from fish markets in Suncheon Bay, South Korea. Crabs with intact claws and appendages were selected and then acclimatized with aeration at 16.0 ± 1.0 °C and a 17:7 h light–dark cycle. They were fed approximately 180 mg of Tetramine (Tetra-Werke, Melle, Germany) daily. The salinity of the water tank was adjusted to 33 ± 1 psu.

### 2.2. Chemicals, Reagents, and Exposure Experiment Design

BPA was purchased from Sigma-Aldrich (99.9% purity; St. Louis, MO, USA) and DEHP was purchased from Junsei Chemical Co. Ltd. (99% purity; Tokyo, Japan). Stock solutions of BPA and DEHP were prepared at 10 mg L^−1^ using acetone as the solvent. These solutions were then diluted to concentrations of 1 µg L^−1^, 10 µg L^−1^, and 30 µg L^−1^ by mixing them with artificial seawater. The crabs were divided into four groups (control and three treatment groups corresponding to 1 µg L^−1^, 10 µg L^−1^, and 30 µg L^−1^; *n* = 180). Each group was sampled for mRNA expression analysis at different time points (1^st^ day, 4^th^ day, and 7^th^ day) after exposure to the three concentrations of BPA and DEHP. The experiment was conducted in triplicates.

### 2.3. Total RNA Extraction and cDNA Synthesis

Total RNA was extracted from the gonads and stomach of control and treated crabs (30–35 mg crab^−1^) using the TRIzol reagent (Life Technologies, Carlsbad, CA, USA) according to the manufacturer’s instructions. Genomic DNA was removed using recombinant DNase I (RNase-free) (Takara, Kusatsu, Japan). The RNA concentration was measured using a Nano-Drop 1000 system (Thermo Fisher Scientific, Waltham, MA, USA), and nuclease-free water (Invitrogen, Waltham, MA, USA) was used to adjust the concentration. RNA integrity was confirmed via 1% agarose gel electrophoresis, and the samples were stored at −80 °C. For single-stranded cDNA synthesis, oligo dT primers (50 μM) and 1 μg of total RNA were used with the PrimeScript^TM^ 1st Strand cDNA Synthesis Kit (Takara, Kusatsu, Japan).

### 2.4. Gene Expression Quantification in M. japonicus

The mRNA expression levels of four candidate genes (*LGBP, Serpin, Tryp, and PE*) in the control and treated crab groups were measured via quantitative real-time polymerase chain reaction (qRT-PCR) (Table 1). The glyceraldehyde-3-phosphate dehydrogenase (GAPDH) gene was used as an endogenous control for relative expression analysis. The qRT-PCR was conducted using AccuPower^®^ 2x GreenStar^TM^ qPCR Master Mix (Bioneer, Daejeon, Korea) in a total volume of 20 μL, which included 5 μL of cDNA diluted 30-fold, 10 μL of 2x SYBR, 0.5 μL of each primer (10 μM), and DEPC-treated water. The qRT-PCR cycle conditions were as follows: 95 °C for 3 min, followed by 40 cycles of 15 s at 95 °C, 35 s at 57 °C, and 20 s at 72 °C, with a final step for melting curve analysis (from 67 °C to 95 °C, with increments of 1 °C every 5 s). Relative gene expression levels were determined by normalizing the expression of the target genes to GAPDH as an internal reference gene using the 2^−ΔΔct^ method [35].

### 2.5. Data Analysis

Statistical significance for all BPA and DEHP treatment groups was determined using R (version 4.3.1). Comparisons with the control group were conducted via one-way analysis of variance (ANOVA) and Student’s *t*-tests. Independent *t*-tests were used to compare the statistical significance of mRNA expression between stomach and gonads in response to different concentrations of BPA and DEHP. Data were represented as mean ± standard deviation.

## 3. Results

### 3.1. Expression of proPO System-Related Genes in Gonads After Exposure to EDCs

**Figure 2:**
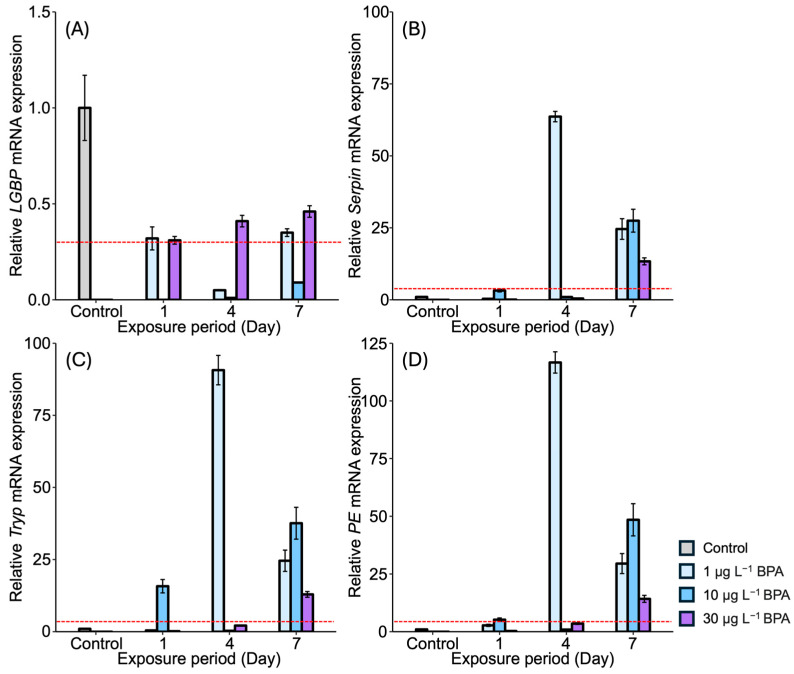
Relative mRNA expression levels of four proPO-related genes in the *M. japonicus* gonads exposed to 1 μg L^−1^, 10 μg L^−1^, and 30 μg L^−1^ BPA for 1, 4, and 7 days, including controls. Data are represented as the mean ± SD. mRNA expression levels of each gene were normalized against *GAPDH*. The red dotted line indicates significantly regulated values compared to the control. (**A**) relative mRNA expression of *LGBP*, (**B**) relative mRNA expression of *Serpin*, (**C**) relative mRNA expression of *Tryp*, (**D**) relative mRNA expression of *PE*.

**Figure 3:**
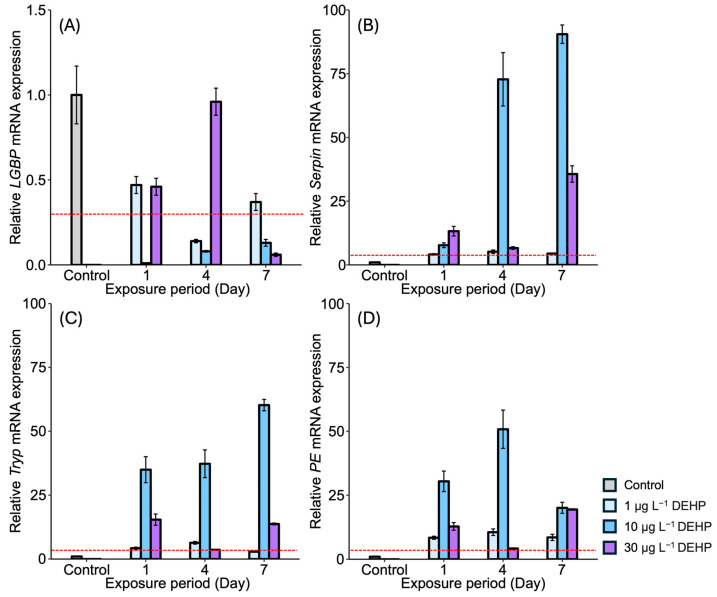
Relative mRNA expression levels of four proPO-related genes in the *M. japonicus* gonads exposed to 1 μg L^−1^, 10 μg L^−1^, and 30 μg L^−1^ DEHP for 1, 4, and 7 days, including controls. Data are represented as the mean ± SD. mRNA expression levels of each gene were normalized against *GAPDH*. The red dotted line indicates significantly regulated values compared to the control. (**A**) relative mRNA expression of *LGBP*, (**B**) relative mRNA expression of *Serpin*, (**C**) relative mRNA expression of *Tryp*, (**D**) relative mRNA expression of *PE*.

The mRNA expression levels of proPO system-related genes were examined in the gonad of *M. japonicus* exposed to BPA and DEHP (Figure 2 and Figure 3). In the gonads, *LGBP* gene expression was significantly reduced compared to the control after exposure to all concentrations of BPA at all time points (Figure 2A). On day 1, the lowest expression of the *LGBP* gene was observed under 10 μg L^−1^ of BPA. The reduced level of *LGBP* gene expression as a result of BPA exposure increased on days 4 and 7. *Serpin* gene expression was generally higher in the gonads of *M. japonicus* individuals exposed to BPA (Figure 2B). On day 4, the highest expression of the *Serpin* gene was observed under 1 μg L^−1^ of BPA (*p* < 0.01). *Serpin* mRNA expression was upregulated under all BPA concentrations on day 7. The expression patterns of the *Tryp* and *PE* genes were similar to that of the *Serpin* gene (Figure 2C,D). On day 1, *Tryp* gene expression increased significantly under 1 μg L^−1^ of BPA, with the highest expression observed in the gonads of *M. japonicus* exposed to this lower concentration. On day 7, *Tryp* gene expression generally increased under all BPA concentrations (Figure 2C). Additionally, the highest expression of the *PE* gene was observed under 1 μg L^−1^ of BPA, with upregulation observed under all BPA concentrations on day 7.

After DEHP exposure, *LGBP* gene expression generally decreased in the gonads of *M. japonicus* over 7 days compared to the control. On day 1, the lowest *LGBP* gene expression was observed under 10 μg L^−^^1^ of DEHP (Figure 3A). On day 7, *LGBP* mRNA expression was mostly downregulated under the higher concentration of 30 μg L^−^^1^ of DEHP. In contrast, other proPO system-related genes, including *Serpin*, *Tryp*, and *PE*, showed significantly increased levels under all DEHP concentrations and across all exposure times (Figure 3B–D). On day 1, *Serpin* gene expression increased in a dose-dependent manner (Figure 3B). On days 4 and 7, the highest *Serpin* expression was observed under a concentration of 10 μg L^−^^1^ of DEHP, with peak expression observed under this concentration on day 7. Similarly, *Tryp* and *PE* were highly upregulated under 10 μg L^−^^1^ of DEHP across all exposure times (Figure 3C,D).

### 3.2. Expression of proPO System-Related Genes in the Stomach After EDC Exposure

**Figure 4:**
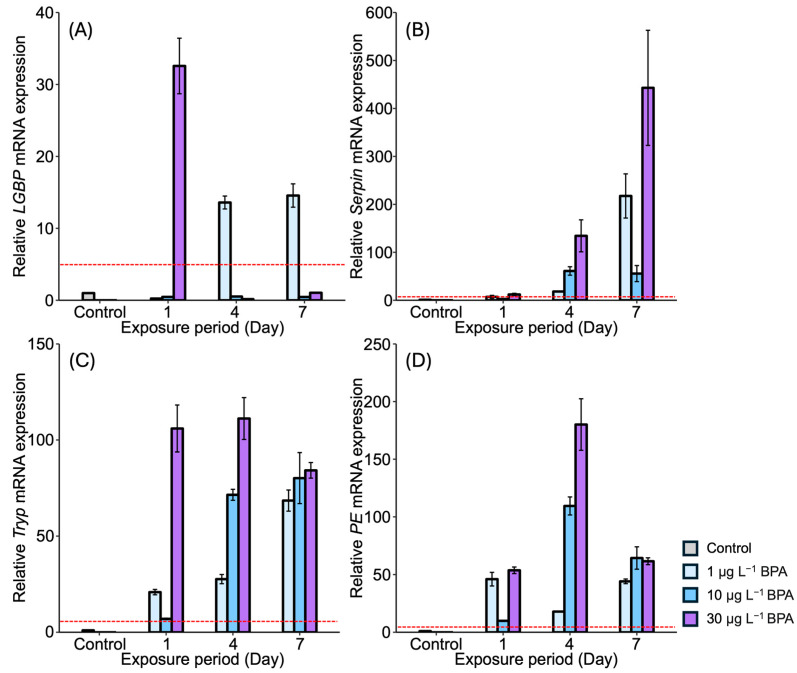
Relative mRNA expression levels of four proPO-related genes in the *M. japonicus* stomach exposed to 1 μg L^−1^, 10 μg L^−1^, and 30 μg L^−1^ BPA for 1, 4, and 7 days, including controls. Data are represented as the mean ± SD. mRNA expression levels of each gene were normalized against *GAPDH*. The red dotted line indicates significantly regulated values compared to the control. (**A**) relative mRNA expression of *LGBP*, (**B**) relative mRNA expression of *Serpin*, (**C**) relative mRNA expression of *Tryp*, (**D**) relative mRNA expression of *PE*.

In the stomach, the *LGBP* gene exhibited the highest expression (32-fold) compared to the controls after 1 day of exposure to the relatively high concentration of 30 µg L^−^^1^ of BPA (Figure 4A). On days 4 and 7, upregulation of the *LGBP* gene was observed under the lowest concentration of BPA, i.e., 1 µg L^−^^1^. However, *LGBP* gene expression decreased under other concentrations and across exposure times. The mRNA expression of *Serpin* generally increased under all BPA concentrations. On day 4, *Serpin* expression increased in an exposure-time-dependent manner, with the highest expression observed under 30 µg L^−^^1^ of BPA after 7 days (Figure 4B). *Tryp* gene expression also increased in the stomach of *M. japonicus* exposed to all concentrations of BPA across all exposure times (Figure 4C). On days 4 and 7, *Tryp* expression increased in an exposure-time-dependent manner. Under the relatively high concentration of 30 µg L^−^^1^ of BPA, *Tryp* gene expression was notably elevated across all exposure times. Additionally, *PE* gene expression was upregulated under all concentrations of BPA across all exposure times (Figure 4D). On day 4, *PE* expression increased in a dose-dependent manner, though the upregulation slightly decreased by day 7.

**Figure 5:**
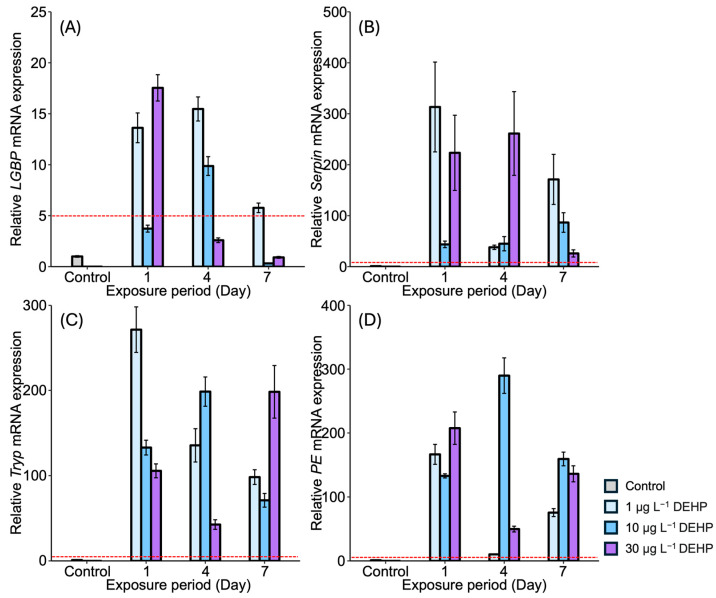
Relative mRNA expression levels of four proPO-related genes in the *M. japonicus* stomach exposed to 1 μg L^−1^, 10 μg L^−1^, and 30 μg L^−1^ DEHP for 1, 4, and 7 days, including controls. Data are represented as the mean ± SD. mRNA expression levels of each gene were normalized against *GAPDH*. The red dotted line indicates significantly regulated values compared to the control. (**A**) relative mRNA expression of *LGBP*, (**B**) relative mRNA expression of *Serpin*, (**C**) relative mRNA expression of *Tryp*, (**D**) relative mRNA expression of *PE*.

After DEHP exposure, *LGBP* gene expression increased across all concentrations after 1 day, with the highest expression (18-fold) observed in the stomach of *M. japonicus* exposed to 30 µg L^−^^1^ of DEHP (18-fold) (Figure 5A). On day 4, *LGBP* gene upregulation was observed across all concentrations of DEHP. However, by day 7, *LGBP* gene expression was downregulated, becoming similar to that of the control after exposure to 10 µg L^−^^1^ and 30 µg L^−^^1^ of DEHP. *Serpin* gene expression generally increased across all DEHP concentrations and exposure times (Figure 5B). On day 1, the highest *Serpin* expression was observed in response to 1 μg L^−^^1^ of DEHP compared to the control. High levels of *Serpin* gene expression were observed in the 30 μg L^−^^1^ DEHP group after 4 days and in the 1 μg L^−^^1^ DEHP group after 7 days. *Tryp* gene expression significantly increased across all concentrations of DEHP and across all exposure times (Figure 5C). The highest *Tryp* expression was observed under 1 μg L^−^^1^ of DEHP on day 1. However, *Tryp* upregulation was not dose-dependent. On day 7, a high level of *Tryp* mRNA expression was observed under the relatively high concentration of 30 μg L^−^^1^ of DEHP. *PE* gene expression reached its highest level on day 4 after exposure to 10 μg L^−^^1^ of DEHP, with *PE* expression being significantly higher compared to the control (*p* < 0.05) (Figure 5D). DEHP exposure generally induced *PE* gene upregulation.

### 3.3. Integrated Biomarker Response (IBR) Index and Heatmap Analysis on M. japonicus Exposed to BPA and DEHP

**Figure 6:**
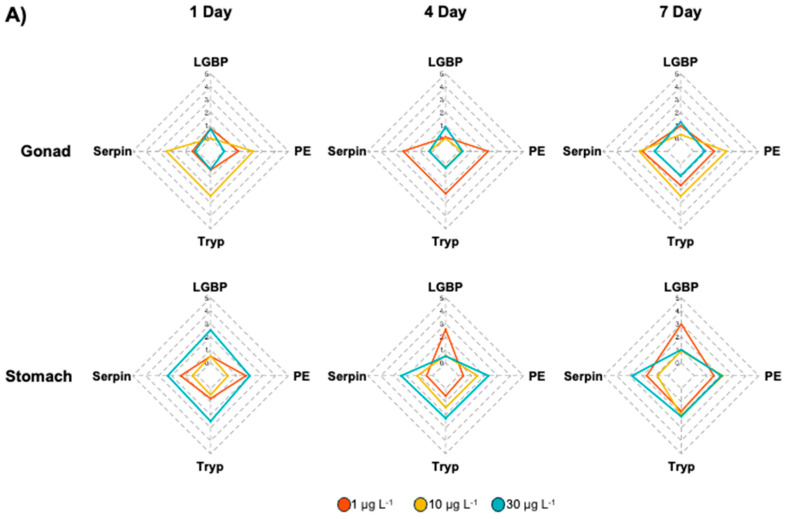

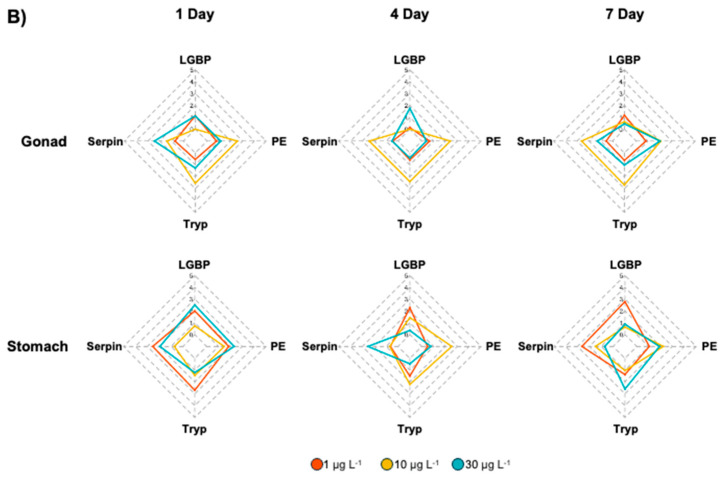
Star plot of IBR values for evaluating gene responses in the *M. japonicus* gonads and stomach exposed to BPA (**A**) and DEHP (**B**).

**Figure 7:**
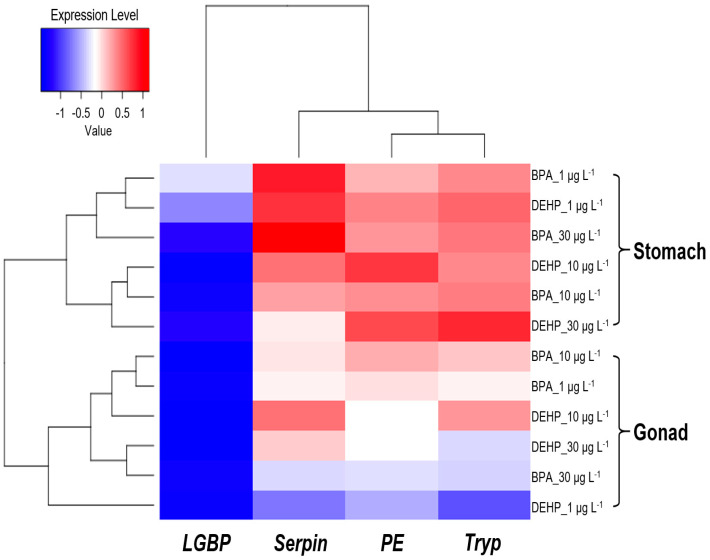
Heatmap of the relative expression levels in the *M. japonicus* gonads and stomach after exposure to 1 μg L^−1^, 10 μg L^−1^, and 30 μg L^−1^ BPA and DEHP on days 1, 4, and 7. Downregulated expression (<0) is indicated in blue, while upregulated expression (>0) is indicated in red. Data from each exposure group were subjected to log transformation.

To identify biomarkers of the toxic effects of BPA and DEHP exposure, IBR indices were calculated based on the expression levels of proPO system genes (Figure 6), after which gene expression patterns were visualized via heatmap analyses (Figure 7). In the BPA-exposed *M. japonicus*, *PE* expression responded to the relatively low concentration of 1 μg L^−^^1^ of BPA in both gonadal and stomach tissues on day 1 (Figure 6A). In the gonads, the *Tryp* gene showed significant upregulation across all exposure times. In the stomach, proPO system-related genes responded sensitively to the relatively high concentration of 30 μg L^−^^1^ of BPA on day 1. On days 4 and 7, *LGBP* gene upregulation was observed under the low concentration of 1 μg L^−^^1^ of BPA, whereas *Serpin* gene expression was higher than that of other genes under the relatively high concentration of 30 μg L^−^^1^ of BPA. In DEHP-exposed *M. japonicus*, all proPO system-related genes exhibited a stronger response in the stomach compared to the gonads on day 1 (Figure 6B). A concentration-dependent increase in *Serpin* IBR values was observed in the gonads 1 day after DEHP exposure, with a decrease in *LGBP* (IBR value: 0.45) on day 7 (Table 2). In the gonads, exposure to relatively high DEHP levels induced *LGBP* gene expression on day 4 and *PE* gene expression on day 7. Under 10 μg L^−^^1^ of BPA, upregulation was observed for the *Serpin*, *Tryp*, and *PE* genes. In the stomach, all genes were generally expressed on day 1, with *LGBP* (2.50) and *PE* (2.31) gene levels being markedly upregulated 1 day after exposure to 30 μg L^−^^1^ of DEHP (Table 2). However, on day 4, different expression patterns were observed under various exposure concentrations, with some genes being markedly upregulated after exposure to particular doses, including the *LGBP* gene in the 1 μg L^−^^1^ DEHP group, *PE* in the 10 μg L^−^^1^ DEHP group, and *Serpin* in the 30 μg L^−^^1^ DEHP group. By day 7, the *LGBP* and *Serpin* genes responded to low levels of DEHP, whereas *PE* (1.91) and *Tryp* (2.60) responded to high levels of DEHP (Table 2).

Our heatmap analyses revealed that the *LGBP* gene exhibited a negative correlation between exposure concentration and tissue, except for the stomach tissues of *M. japonicus* exposed to 1 μg L^−^^1^ BPA and DEHP. In contrast, the *Serpin*, *Tryp*, and *PE* genes displayed a positive correlation in the stomach (Figure 7). In *M. japonicus* crabs, proPO system-related genes responded more sensitively in the stomach than in the gonadal tissue. The *Tryp* gene expression was highly upregulated in the gonads after exposure to both BPA and DEHP. In the stomach, gene expression was more sensitively influenced by DEHP exposure compared to BPA exposure on day 1. However, the effects of BPA and DEHP on gene expression patterns were significantly different on days 4 and 7.

## 4. Discussion

Marine and coastal ecosystems serve as vast aquatic storage systems, acting as major sinks for pollutants originating from landscapes and freshwater sources [36]. These ecosystems have been heavily impacted by human activities, leading to elevated levels of various contaminants in marine sediments [37]. EDCs such as BPA and DEHP enter marine and coastal ecosystems primarily through effluents from wastewater treatment facilities (WWTF). These chemicals have been ubiquitously detected in marine biota, as well as in surface waters and sediments across all environments [37]. EDCs are exogenous substances that can mimic or interfere with the endocrine system, causing adverse effects in organisms and altering critical biological processes, including reproduction, growth, organ development, metabolism, immunity, and behavior [19,38,39,40]. In crustaceans, EDCs can disrupt the molting process, which is vital for their growth and development [41]. Recent studies have shown that exposure to BPA or DEHP induces oxidative stress and alters the expression of inflammation-related genes and calcium ion homeostasis genes in crabs [41,42]. In this study, we investigate the expression of immune-related genes in the stomach and gonads of *M. japonicus* following DEHP exposure and observe a significant increase in immune-related gene expressions in response to toxic effects. Previous studies linking DEHP to immunity have shown that exposure to DEHP interferes with immune functions in a variety of aquatic species by altering levels of inflammatory factors, as shown by its effects on phagocytosis in the common carp [43]. We also observe that DEHP exposure results in concentration- and duration-dependent differences in gene expression, which differed from the general trend of increasing toxic effects in a concentration-dependent manner. The structural and functional specificity of EDCs may be responsible for low-dose effects, where a concentration-dependent toxicity trend is not exhibited [44,45]. In the zebrafish and *Cyprinus carpio* L., DEHP exposure has been shown to yield inconsistent effects on innate immune gene expression, highlighting its potential to disrupt immune-related metabolic pathways in vivo [43,46]. Moreover, DEHP exposure is linked to impaired humoral defense mechanisms, ultimately diminishing the effectiveness of immune responses against pathogens [47,48,49]. BPA exposure has been associated with delayed gonad development, metabolic disturbances, and endocrine disruptions in Pacific whiteleg shrimp [50]. Additionally, BPA exposure has been shown to decrease the proportion of red granulocytes in *Tegillarca granosa*, leading to reduced phagocytic activity and impaired reactive oxygen species (ROS) production, both of which are vital for effective immune responses [51]. This reduction in red granulocytes and ROS generation compromises essential blood cell functions, ultimately weakening the organism’s immune defense [52,53]. In marine environments, plastic materials, including plasticizers such as DEHP, BPA, and dibutyl phthalate (DBP), frequently attach to microplastics, creating a combination that is highly toxic to marine life [54]. In view of the evidence indicating that DEHPs can disrupt the functioning of cells within the immune system and impede crucial immune pathways, it is imperative that regulatory action and continuous monitoring of these contaminants in aquatic ecosystems be carried out in order to protect marine biodiversity and the overall health of these ecosystems.

The potential effects of EDC exposure have been identified at the molecular level in *M. japonicus*, an indicator species in coastal sediment environments. Exposure to DEHP or BPA has been found to induce alterations in the *p38* mitogen-activated protein kinase (*MAPK*) gene, which plays a crucial role in cellular immune and apoptotic pathways [55]. Both exposures also resulted in changes to the histological structure and the expression of immune neurotransmitter-related genes in *M. japonicus* [56]. The innate immune system, which serves as the first line of defense to eliminate invading pathogens, is essential for regulating the activation of immune cells and maintaining control over antioxidant defense, inflammatory responses, and tissue homeostasis [56,57]. The cellular stress caused by immune disruption may ultimately affect epigenetic responses across generations, as innate immune cells and inflammatory processes are tightly regulated by epigenetic mechanisms [58]. *Serpin* plays a significant role in regulating immune pathways in arthropods, potentially serving as a modulator that transiently enhances immune responses in the presence of toxic substances [59]. Inhibitory effects on enzyme activities during digestion and pathogen degradation in *Chironomus riparius* have been documented [60]. The present study indicates that alterations to *Trypsin* and *Serpin* expression in mud crabs exposed to EDCs may signify immune system and digestive dysfunction. Peroxinectin is an immune component that plays a crucial role in cell adhesion and peroxidase activity, both of which are essential for the elimination of invading microorganisms and pathogens in invertebrates [60]. The increase in peroxinectin expression suggests a defensive response to external invasion, a finding consistent with the results of previous studies in crustaceans [61,62]. ProPO is an important enzyme in the innate immune system of crustaceans and is involved in the melanization reaction [63]. Activation of the proPO system ultimately results in a melanization response in the exoskeleton of invertebrates [64,65]. Moreover, the elevated expression of peroxinectin is associated with the generation of reactive oxygen species, which can lead to oxidative stress [66,67]. The observed increase in peroxinectin expression in this study suggests that DEHP exposures induce a protective effect against oxidative stress by stimulating the immune system of the mud crab, ultimately resulting in a melanization response as the external damage.

In a previous study, we reported that exposure to these EDCs alter the expression of proPO system-related genes in *M. japonicus* [8]. The present study further demonstrates that exposure to EDCs, such as BPA or DEHP, induces different expressions of innate immune proPO system-related genes depending on the tissue type in *M. japonicus*. In the gonads, which are associated with reproductive function, low-level exposure to BPA or DEHP primarily triggers a transcriptional response in the *Tryp* gene among the proPO system-related genes tested. In the stomach, which is responsible for digestive functions, early exposure to DEHP causes all proPO system-related genes to respond across all concentrations, while BPA exposure elicits a response mainly at higher concentrations. At later exposure times, low-level exposure to BPA or DEHP induces a transcriptional response in the *LGBP* gene within the stomach of *M. japonicus*. These findings suggest that the immune defense mechanisms against toxicant exposure may vary between tissues with different functions.

## 5. Conclusions

In this study, we observe changes in the mRNA expression of genes related to proPO system activation in the gonads and stomach of *M. japonicus* exposed to the EDCs BPA and DEHP. BPA exposure results in the upregulation of *Tryp*, *Serpin*, and *PE* in gonadal tissue, while it downregulates the *LGBP* gene. On day 1, the lowest expression of the *LGBP* gene in the gonads is observed under 10 μg L^−1^ of both BPA and DEHP. However, in the stomach, *LGBP* gene expression becomes upregulated following BPA exposure. The expression patterns of *LGBP* differ depending on tissue type. The expression levels of the *Serpin*, *Tryp*, and *PE* genes become elevated across all concentrations of both EDCs compared to the control group. In the gonads, *Serpin* and *Tryp* become upregulated in a time-dependent manner under 10 μg L^−1^ of DEHP. In the stomach, exposure to either BPA or DEHP induces a higher expression of proPO system-related genes compared to the control group on day 1 across all concentrations. The transcriptional response to DEHP is generally higher than that to BPA. These results highlight distinct expression patterns depending on tissue type and EDC concentration. Moreover, our findings suggest that EDC exposure disrupts the immune defense system via the regulation of proPO system-activating genes.

## Figures and Tables

**Table 1:** Primers used for proPO-related gene amplification in this study.

Gene	Primer Sequence (5′–3′)	Amplicon Size (bp)	Efficiency (%)	Accession Number
LGBP_F	AATGGCTTCTTCCCTGACGG	131	100.0	KJ653260
LGBP_R	CTGATCTTGCCCTCACCCTG			
Serpin_F	TTTGGAACGTGGGAGTATGC	74	93.0	MH41109
Serpin_R	TGCACATTGGGAATCGCATG			
Tryp_F	CCTAGAGGTCGGGGTCAAGA	91	99.5	KJ653261
Tryp_R	CCTATCCAGCTCGAGCAGTG			
PE_F	CTGACCACCATACACACGCT	98	90.0	KF804082
PE_R	TGGAACACTTGCTCGTCCTG			
GAPDH_F	TGCTGATGCACCCATGTTTG	147	102.5	KJ653265
GAPDH_R	AGGCCCTGGACAATCTCAAAG			

**Table 2:** IBR index for evaluating gene responses in gonadal and stomach tissues of *M. japonicus* exposed to 1 μg L^−1^, 10 μg L^−1^, and 30 μg L^−1^ BPA and DEHP.

Chemical	Organ	Concentration (μg/L)	proPO System-Related Gene					IBR Value
			*LGBP*	*Serpin*	*Tryp*	*PE*	Mean	
BPA	Gonad	Control	2.67	0.00	0.15	0.20	0.75	2.45
		1	0.96	1.96	1.65	1.60	1.54	1.17
		10	0.29	2.20	2.48	2.53	1.87	4.31
		30	1.25	1.03	0.91	0.85	1.01	2.61
	Stomach	Control	1.00	0.58	0	0.05	0.41	0.46
		1	2.98	1.67	1.74	1.52	1.98	1.38
		10	0.92	0.85	2.04	2.22	1.51	3.90
		30	1.01	2.81	2.14	2.12	2.02	4.24
DEHP	Gonad	Control	2.65	0.46	0.56	0	0.92	2.58
		1	1.17	0.54	0.63	0.82	0.79	1.35
		10	0.63	2.62	2.69	2.08	2.00	1.93
		30	0.45	1.29	1.02	2.01	1.19	3.78
	Stomach	Control	0.90	0.37	0.18	0	0.37	0.34
		1	2.79	2.62	1.37	1.05	1.96	1.31
		10	0.64	1.50	1.04	2.24	1.35	3.40
		30	0.87	0.70	2.60	1.91	1.52	2.97

## Data Availability

The data in this study will be disclosed upon request.
